# Unlocking the enigma: Combined percutaneous-transhepatic and endoscopic strategies for retrieval of severed Dormia basket in choledocholithiasis. A case report and literature review

**DOI:** 10.1016/j.radcr.2024.03.074

**Published:** 2024-04-19

**Authors:** Mohammed Misbahuddin-Leis, Muzaffer Ankolvi, Manisha Mishra, Krisztina Dubasz, Aleksander Marinov, Thomas Müller, Christian Graeb, Boris Radeleff

**Affiliations:** a Medical Faculty Heidelberg, Heidelberg University; b Department of Diagnostic and Interventional Radiology, Sana Klinikum Hof GmbH, Academic Teaching Hospital of the Friedrich-Alexander-University Erlangen-Nuremberg; c Department of Gastroenterology, Hepatology, Infectiology, Hematology and Oncology, Sana Klinikum Hof GmbH, Academic Teaching Hospital of the Friedrich-Alexander-University Erlangen-Nuremberg; d Department of Visceral and Abdominal Surgery, Sana Klinikum Hof GmbH, Academic Teaching Hospital of the Friedrich-Alexander-University Erlangen-Nuremberg

**Keywords:** IR, ERCP, Impacted basket, Goose neck snare, Lithotripsy, Rendezvous technique

## Abstract

Choledocholithiasis, characterized by the presence of stones in the common bile duct, poses significant challenges in clinical management, particularly when the stones are massive. While endoscopic methods are often effective in stone removal, complications such as the impaction of foreign bodies like Dormia baskets can occur. These complications may necessitate alternative approaches, including surgical intervention, highlighting the importance of exploring innovative interventional techniques. We report on an 89-year-old patient presenting with massive choledocholithiasis, involving complete filling of the intra- and extrahepatic bile duct system with large stones up to a maximum of 2 cm. The patient underwent interventional removal of a Dormia basket (3.5Fr. Boston Scientific, USA) impacted in the common bile duct. This procedure proved challenging due to the metallic end marker of the basket perforating through the wall of the distal common bile duct, rendering it fixed. Given the complexity of the case, a parallel approach combining percutaneous transhepatic cholangiography and drainage with simultaneous endoscopy was employed to successfully extract the fixed Dormia basket. In cases of severe choledocholithiasis complicated by the impaction of foreign bodies such as Dormia baskets, innovative interventional strategies are crucial for successful management. Our case highlights the effectiveness of a parallel approach involving percutaneous transhepatic cholangiography and drainage alongside simultaneous endoscopy in safely removing the fixed foreign body from the common bile duct. This multidisciplinary approach not only offers a viable alternative to surgical intervention but also underscores the importance of collaboration between interventional radiologists and endoscopists in optimizing patient outcomes in complex biliary interventions.

## Introduction

Endoscopic retrograde cholangiopancreatography (ERCP) is the gold standard in the treatment of choledocholithiasis. Stones in the common bile duct (CBD) are successfully removed in 85%–95% of cases (after optional sphincterotomy) using a dormia basket (DB) or balloon catheter [1]. For stones larger than 1 cm, additional calculus-reducing methods are usually used, such as mechanical lithotripsy, electrohydraulic probe lithotripsy (EHL), extracorporeal shock wave lithotripsy (ESWL) or laser lithotripsy. The most widespread method is mechanical lithotripsy, in which the stone is caught using DB and defragmented using shock waves. In most clinical studies, the procedure has proven to be highly efficient, with published technical success rates of 90% to 97% [2].

Complications arising from the use of a Dormia or lithotripter basket occur in approximately 0.8% to 6% of procedures. This usually involves an impaction of the Dormia/lithotripter basket with a pre-existing bile duct stone in the common bile duct (CBD) or when one or more traction wires break during the mechanical lithotripsy procedure [3]. The removal of an impacted Dormia/lithotripter basket is mandatory in order to avoid, among other things, cholangitis and injury to the bile ducts or intestines caused by the metallic foreign body.

We report on the successful interventional removal of a CBD-impacted DB (3.5Fr. Boston Scientific, USA) in an 89-year-old patient with massive choledocholithiasis, characterized by complete filling of the intra- and extrahepatic bile duct system with large stones up to a maximum of 2 cm with the fractured wire protruding outside of the oral cavity during an attempt to retrieve the stones. To address this challenge, we employed a parallel approach involving percutaneous transhepatic cholangiography and drainage (PTCD) alongside simultaneous endoscopy (to cut the retaining wires), allowing for the successful and complete removal of the DB fixed in the CBD.

## Case report

We report on an 89-year-old Caucasian female patient who tested positive for COVID-19 at the time of the procedures and who presented to our emergency department without any known previous illnesses after sudden, repeated bilious vomiting and stool retention with a known tendency to constipation.

Clinically, a distended abdomen with tenderness throughout the abdomen without rigidity was noted. Laboratory chemistry showed increased inflammation and cholestasis parameters (leukocytes 20 GPt/l (4-10 Gpt/l), CRP 188 mg/L (<5mg/L), serum bilirubin: 2.35mg/dL (<0.2 mg/dL), Gamma-GT: 87.6 U/L (up to 39 U/L) and ALP: 121 U/L (<187 U/L.)).

**Fig. 1:**
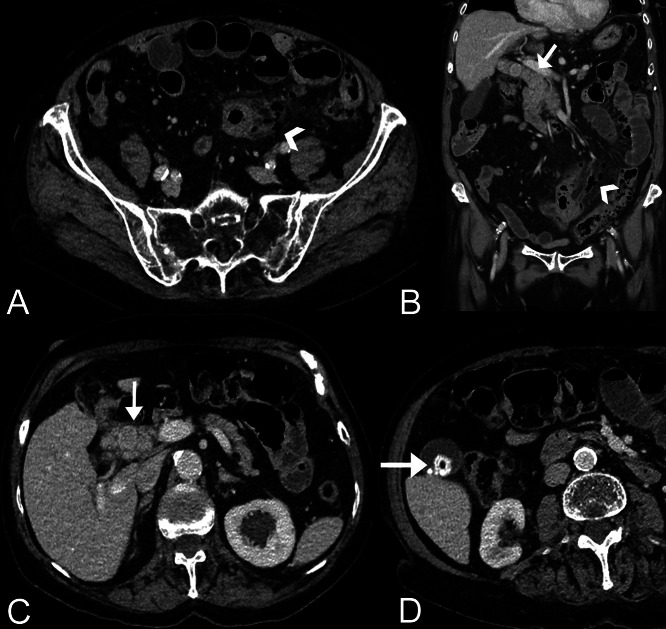
**(**A-D) (A and B) Contrast-enhanced CT in transversal and coronal reconstruction reveals Meckel diverticulitis (white arrowhead) situated in the distal jejunum (left abdomen), accompanied by local peritonitis and numerous surrounding extraluminal gas bubbles indicative of perforation. Additional findings include signs of subileus. (C and D) Cholelithiasis is also depicted, with marked choledocholithiasis (white arrows).

Abdominal sonography revealed multiple echodense concrements in the CBD, particularly in the distal third, as well as small intestinal loops dilated to approximately 3 cm with pendulum peristalsis. In the contrast-enhanced (70 mL from Accupaque, GE Healthcare, USA) multiphase contrast MDCT examination (Revolution HR, GE Healthcare, USA) of the abdomen, which was carried out immediately afterwards for further clarification, the pronounced cholecystolithiasis and choledocholithiasis were confirmed (see Fig. 1B-D). It showed multiple, packed hyperdense concrements measuring up to 18 mm throughout the DHC with consecutive intrahepatic cholestasis emphasizing the left lobe of the liver. Analogous to the ultrasound, there was massive wall thickening of a 10 cm long segment of the small intestine (distal jejunal loop in the left middle abdomen) with fat stranding and evidence of several extraluminal air bubbles (Fig. 1A and B).

**Fig. 2:**
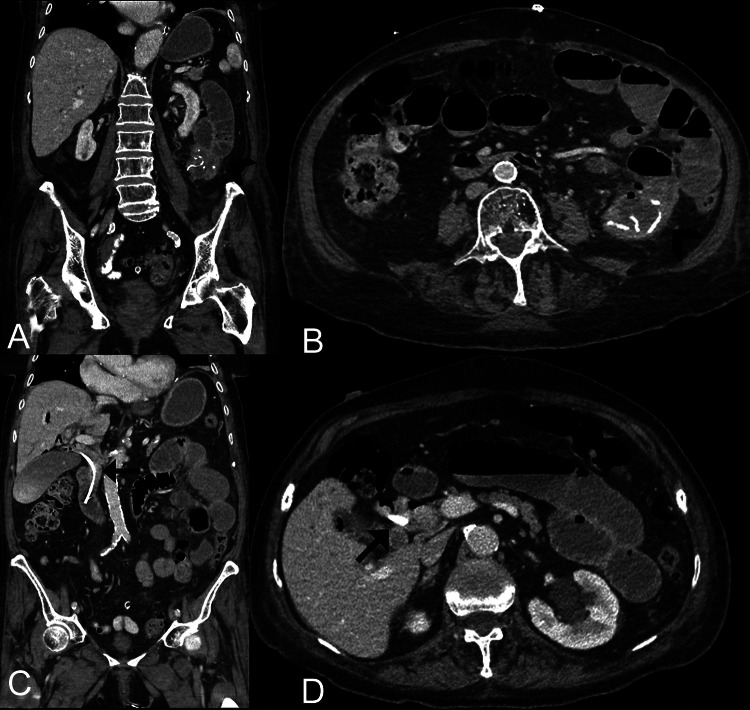
**(**A-D) **(**A and B) Contrast-enhanced follow-up CT following jejunum segment resection (black arrowhead) and after ERCP without signs of any postoperative complication. (c and d) depiction of the correct placed plastic endoprosthesis in the CBD (black arrows) after ERCP, although signs of subileus persist.

With radiological suspicion of a perforated Meckel's diverticulum, the patient underwent an emergency exploratory laparotomy following the CT scan. The radiological suspicion was confirmed, a jejunal segment resection was performed with removal of the perforated Meckel's diverticulum. Postoperatively, a massively distended abdomen developed with the clinical picture of paralytic subileus. A CT follow-up examination carried out on the same day confirmed this suspicion, without evidence of an abscess or other postoperative complications (see Fig. 2A-D).

**Fig. 3:**
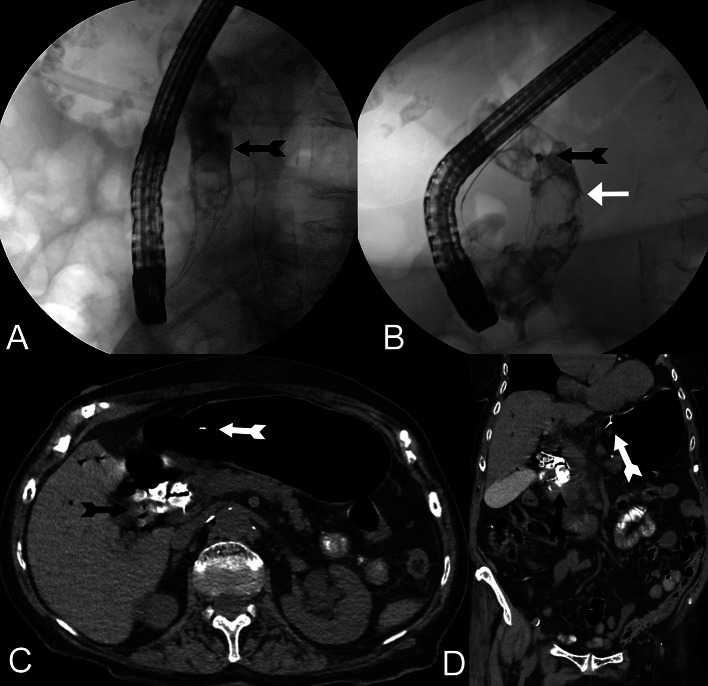
**(**A-D) **(**A and B) Second ERCP with the attempt to disintegrate and/or retrieve multiple bile stones in the CBD. Unfortunately, the gastroenterologists encountered a rupture of the DB (black feather arrow) with the pulling-wire (white arrow) (c and d) Contrast-enhanced follow-up CT after the second ERCP showed the severed DB (black feather arrow) impacted in the CBD with the pulling-wire reaching from the DB through the complete nasopharyngeal tract (white feather arrow).

After the clinical symptoms improved over time, ERCP was performed under deep analgesic sedation using propofol (cumulative 190 mg) 2 days after the operation. Parallel to the findings in the CT scan, multiple large stones were identified in the CBD, accompanied by the presence of a juxtapapillary duodenal diverticulum. Following papillotomy, a 10 Fr Plastic endoprosthesis was introduced into the CBD, (type: straight/duodenally curved, stent length 8 cm, diameter 3.3 mm). In the later second ERCP session (now 35 days after the first ERCP or 37 days postoperatively), again under deep analgesic sedation using propofol, the plastic endoprosthesis was removed and an attempt was made at electrohydraulic lithotripsy with the EHL probe (Walz Elektronik GmbH, Germany), which was not very effective due to the partly very hard stones. During the subsequent attempt at mechanical lithotripsy using a DB, the pulling wire tore extracorporeally on the twist handle, so that the DB and the trapped stones got stuck in the CBD (see Fig. 3A and B). In this situation, a new attempt was made using lithotrypsy, i.e. the fragmentation of stones within the impacted DB using an EHL probe, which proved to be less than successful. Despite several attempts, the DB could not be removed endoscopically and was pulled into the papillary as the papilla became increasingly lacerated. There, two of the four retaining wires could be severed using an OTSC cutter (Ovesco Endoscopy AG, Germany). The DB ultimately could not be removed (due to the large stones trapped in the DB).

**Fig. 4:**
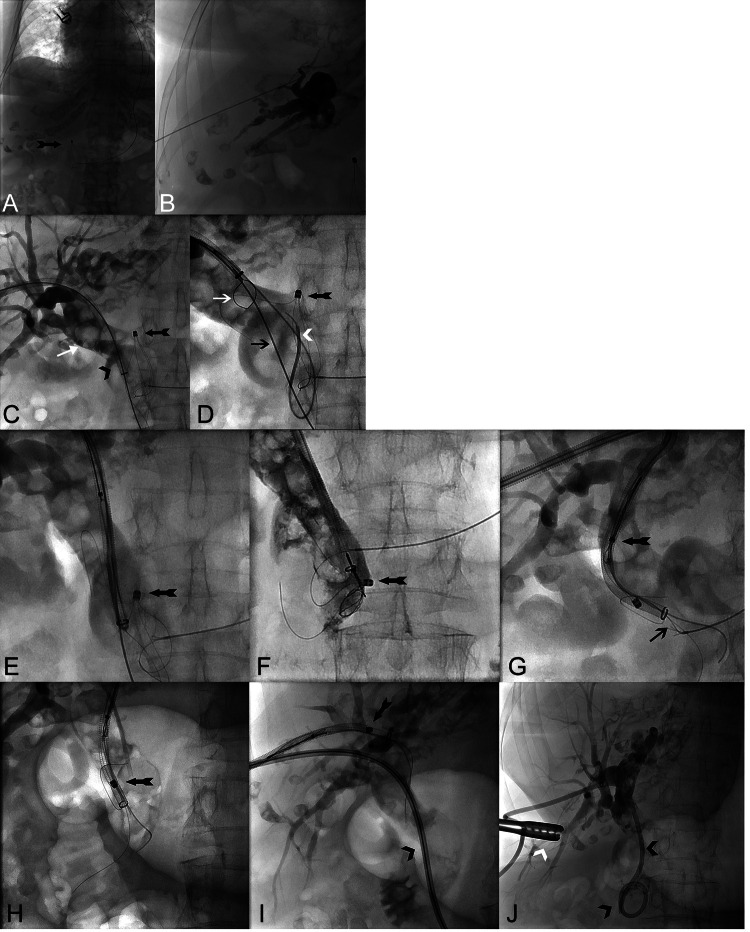
(A-D) (A) Severed DB (black arrow) impacted in the CBD with the pulling-wire. (B) Ultrasound-guided percutaneous transhepatic biliary approach (Neff Percutaneous Access Set™, Cook, USA) into the right-sided biliary system in the angio-suite. (C and D) Following the insertion of a 10Fr sheath (Arrow®, Teleflex, USA, black arrow head), recanalization into the duodenum was achieved using a 0.035′ wire (Radifocus™, Guide Wire, Terumo, Japan) and a 4F catheter (Radifocus™, Glidecath™, Terumo, Japan, white arrow head). An 0.035′ heavy-duty wire was subsequently placed as a support wire (Amplatzer wire, BSCI, USA, black arrow) before attempts were made to capture the DB. The imaging reveals intrahepatic bile ducts containing several small and larger stones (white arrow), with the DB (black feather arrow) situated in the CBD. Unfortunately, the metallic tip of the DB had already perforated the CBD. The combination of the DB tip perforation and the presence of large concrements inside the DB elucidates why the endoscopic removal proved unsuccessful. (E-G) **(**e and f) To mobilize the DB and its metallic tip embedded in the CBD wall, various goosenecks (Amplatz™, Medtronic, USA) in three different sizes and versions with one to three loops were attempted. However, only through the combination of a 4F Catheter (INFINITI®, Cordis™, USA) and an EN-snare endovascular system (ONE Snare®, Merit Medical, USA), the successful mobilization of the DB at the distal end and its retraction into the 10F sheath was achieved (black feather arrow). (g) The final two remaining holding wires connected to the DB were endoscopically cut (black arrow). (H-J) (H and I) The DB, snared by the gooseneck, was successfully drawn entirely into the 10F sheath (black feather arrow). The sheath was then completely removed through the percutaneous transhepatic approach. (J) With the help of a large tong (white arrow head) and in it folded 3 sterile pieces of gauze, we were able to close the access channel after 15 minutes of manual compression. Because of the large 10F approach and because of the perforation of the CBD by the tip of the basket, a new 8.5F PTBD (black arrowhead) over the right-sided bile duct was placed.

**Fig. 5:**
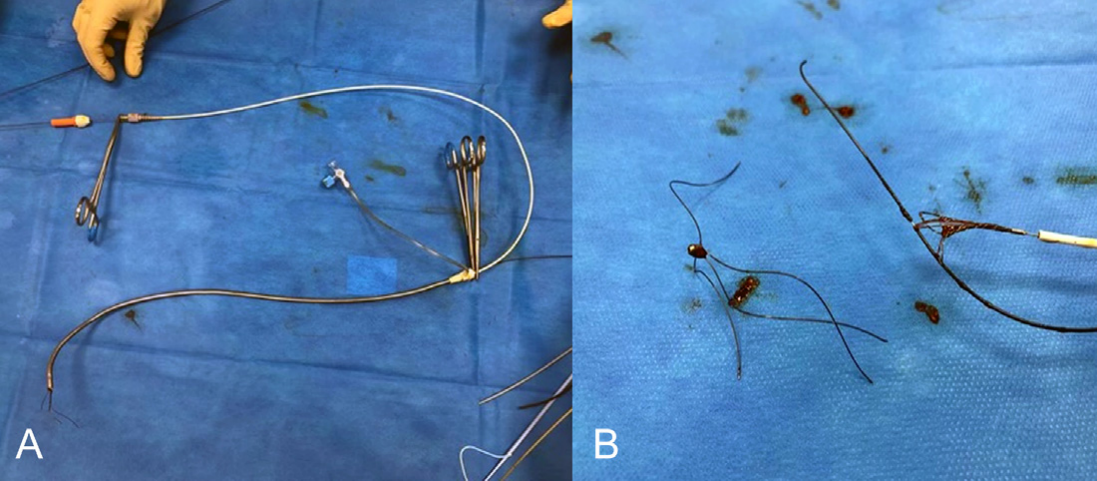
(A and B) Images of the saved DB. (A) the removed sheath with a still retracted DB. (B) after removal of the DB out of the sheath.

In the interdisciplinary discussion that immediately followed (visceral surgery, gastroenterology and interventional radiology), it was decided to attempt to salvage the DB using an interventional radiological approach. A CT examination carried out on the same day showed evidence of a small amount of free air around the portal vein and the hepatic hilum, but no contrast medium leakage, so that it was assumed that the perforation of the CBD was concealed by the DB (Fig. 3C and D). In light of this, the decision was made to forgo the insertion of a completely self-expandable metal stent (SEMS) and instead opt for interventional retrieval of the DB. The next day (38 days postoperatively), the ultrasound-guided puncture of the right biliary system was performed under general anesthesia in our angio-suite using the Neff set (Percutaneous Access Set™, Cook, USA). After contrast medium injection, severely congested bile ducts with multiple concretions were seen in the CBD, which extended into the intrahepatic bile ducts (Fig. 4A and B). The DB was located in the distal third of the CBD with the metal tip of the basket penetrating its lateral wall. After insertion of a metal-coated 10Fr sheath (Arrow®, Teleflex, USA), the CBD was recanalized into the duodenum using a 0.035′' wire (hydrophilic guide wire with curved tip, Terumo, Japan) and a hydrophilic coated 4Fr. catheter (Glidecath™, Terumo, Japan) (Fig. 4C). To mobilize the DB fixed in the CBD, various goosenecks (Merit Medical, USA) in 3 different snare sizes (10, 15, and 20 mm) and with a single or three snares were used (Fig. 4D). The mobilization of the DB at the distal end of the CBD and subsequent withdrawal into the 10Fr sheath proved technically feasible only through the utilization of a shepherd's crook-shaped 4Fr. Simmons catheter (INFINITI®, Cordis™, USA) in conjunction with a 4Fr. EN Snare endovascular system (ONE Snare®, Merit Medical, USA) featuring 3 loops (Fig. 4E-G). Due to the last two remaining holding wires (the other 2 holding wires had already been severed during the ERCP), the DB could not initially be removed through the percutaneous sheath even after capture. However, the gastroenterology colleague managed to cut the two still intact holding wires of the DB directly in the angio-suite using the OTSC cutter (Ovesco Endoscopy AG, Germany). Immediately after cutting the 2 holding wires, the bundle of material consisting of the DB and capture system, which had now been completely pulled into the sheath, could be recovered by removing the 10Fr access sheath, i.e. the percutaneous access had to be completely abandoned (Fig.4H-I). Immediately after removal of the sheath, the access channel and the former sheath entry point were closed with 15 minutes of tightened compression of the percutaneous access using large forceps and 3 sterile pieces of gauze folded into it (Fig. 4J). Due to the CBD perforation caused by the tip of the now recovered DB, a second PTCD system was introduced into the right-sided bile duct system. Following the successful placement of an 8.5 Fr. PTCD as internal-external drainage, the procedure was concluded (Fig. 4, Fig. 5). The patient was finally discharged 12 days after DB extraction in a stable general condition (leukocytes: 11 GPt/l (4-10 Gpt/l), CRP: 40 mg/L (<5 mg/L), serum bilirubin: 0.6 mg/dL (<0.2 mg/dL), Gamma-GT: 112 U/L (up to 39 U/l) and ALP: 121 U/L (<187 U/l).

## Discussion

ERCP is the standard treatment for choledocholithiasis. As described in the introduction, a high percentage of bile duct stones are successfully removed after (optional) sphincterotomy and mechanical lithotripsy using a DB or balloon catheter [4], [5], [6]. Stone impaction, stone size, and the ratio of stone size to bile duct diameter (greater than 1) have been recognized as predictive factors for the failure of mechanical lithotripsy procedures. These factors have been emphasized in multiple studies examining the predictors of unsuccessful mechanical lithotripsy for large bile duct stones [7]. Typical and frequent post-interventional complications include pancreatitis (5%), bleeding (2%), infections (2.3%) and even perforation of the duodenum or bile duct (1%–2%) [5].

In contrast, the impaction of a DB used to treat choledocholithiasis is a rare complication and is usually due to either blockage of the DB due to a large trapped calculus in the DB or a break in the puller wire. This has been reported in approximately 0.8%-6% of procedures during mechanical lithotripsy [1,8]. Leaving an impacted or even completely torn DB in the bile ducts is associated with relevant potential complications, including cholangitis, pancreatitis or even lesions of the bile ducts or intestines. Therefore, timely removal of the affected DB is essential as any delay in treatment may increase the severity of complications [5,9]. Moreover, laceration of the papilla can be of significant concern during the removal of the DB which can be prevented by avoiding extra force, manipulation und overstretching the wires.

To analyze the technology used to remove DBs, we conducted a literature search regarding the recovery of demolished DBs between the years 1968 and 2023. There were 55 publications in the literature with 124 cases of DB left behind. According to the literature, endoscopic techniques are the most frequently used methods for retrieving DBs that remain in the body (approx. 80%), e.g. by retrieving them through a second DB, if necessary after extending the sphincterotomy, shattering the stone fixing the DB using laser lithotripsy or mechanical rescue lithotripter. These endoscopic techniques for retrieving the DB are complex and dependent on the individual expertise of the gastroenterologist. In approximately 20%, salvage was carried out using surgical procedures (e.g. laparoscopic extraction, as first described in 2000 by Ainslie et al. [6]). However, these are not available in every hospital, which may lead to delays due to the patient having to be transferred.

Alternative therapeutic options for the removal of severed Dormia basket fragments include extracorporeal shockwave lithotripsy, balloon dilatation, a second Dormia basket, rescue mechanical lithotripter, exchange of metal wires, exchange of metal sheaths, extension of sphincterotomy, laser lithotripsy, surgical intervention with options including open or laparoscopic exploration of the bile ducts and conservative management tailored to the individual patient's needs and clinical scenario.

There have been case reports, such as that by Sheridan et al. [10], describing the management of lithotripter basket impaction using percutaneous transhepatic intracorporeal electrohydraulic lithotripsy. Additionally, Halfhide et al. [11] employed percutaneous transhepatic choledochoscopic release of an impacted basket after stone fragmentation by electrohydraulic lithotripsy. These cases exemplify the use of percutaneous choledochoscopic lithotripsy to fragment impacted biliary stones from the perspective of interventional radiologists.

In our case, despite the bilestone measuring 2 cm in size, the decision to proceed with ERCP for stone removal was made following emergency exploratory laparotomy for Meckel's diverticulum and jejunal segment resection. Considering this and the associated risks of another surgical intervention for the elderly patient, ERCP was deemed a more suitable option, with the aim of minimizing morbidity and mortality. Given the clinical high risk of complications, the availability of experienced endoscopists, and adequate resources for safe ERCP for large stones, the decision was further supported. Informed consent was obtained from the patient after thorough discussion of associated risks. This decision was reached following a comprehensive assessment, taking into account factors such as overall health status, presence of complications, and patient preferences.

Since endoscopic salvage was unsuccessful, we decided then to use a combination of PTCD and simultaneous endoscopy due to the further potential morbidity (5%-10%) and mortality (1%) associated with surgical salvage [12]. This Rendezvous Technique, involving coordinated efforts between interventional radiology and endoscopy teams, offers a valuable approach in overcoming technical challenges during complex biliary interventions [13,14]. While the conventional technique allows for a synergistic application of endoscopic and radiological interventions to access and extract stones from the biliary system, we strategically employed this approach to successfully remove a DHC-impacted DB. The technique was first introduced by Fujita et al. published in 1988, who successfully removed a DHC-impacted DB including trapped stones using combined endoscopic sphincterotomy and percutaneous transhepatic cholangioscopic lithotripsy [9]. Kwon et al. In 2011, published a case of a successful, purely percutaneous interventional retrieval of a DB system remaining in the DHC using an Amplatz goose-neck snare [15]. In this case, we worked using a combined percutaneous transhepatic and endoscopic approach (to cut the last 2 intact retaining wires) - this is the first report describing such a technique.

## Conclusion

The combination technique using percutaneous transhepatic and endoscopic access enabled successful removal of an impacted torn DB from the CBD including the broken puller wire without complications and should be considered as an alternative treatment method for patients with an impacted DB and a broken puller wire when endoscopic attempts have failed to avoid the risks of open surgery.

## Patient consent

I state that written and informed consent was taken from the patient for publication of this case. The patient was informed that no personal details will be revealed in the publishing of this case.
